# Taking embodiment seriously in public policy and practice: adopting a procedural approach to health and welfare

**DOI:** 10.1007/s40592-023-00183-x

**Published:** 2023-11-04

**Authors:** Joseph T F Roberts

**Affiliations:** University of Birmingham, Birmingham, UK

**Keywords:** Embodiment, Medical practice, Health Policy, Participation, Epistemic injustice

## Abstract

It is a common refrain amongst phenomenologists, disability theorists, and feminist legal theorists that medical practice pays insufficient attention to people’s embodiment. The complaint that we take insufficient account of people’s embodiment isn’t limited to the clinical interaction. It has also been directed at healthcare regulation and welfare policy. In this paper, I examine the arguments for taking embodiment seriously in both medical practice and welfare policy, concluding we have good reasons to take better account of people’s embodiment. I then set out two challenges to taking embodiment seriously in public policy. First, given the amount of variation in how people are embodied, there is strong possibility that adjusting policy to benefit particular individuals based on an appreciation of their embodied experiences could be detrimental towards other individuals. The second challenge concerns how to ensure that people’s testimony about their first-person embodied experience is subject to adequate scrutiny without this resulting in epistemic injustice. I argue that the solution to both of these challenges is to devise a just procedure for soliciting people’s testimony and taking it into account in the policy development process. As such, I also provide an outline of what a just procedure should look like.

**Supplementary Information:**

The online version contains supplementary material available at 10.1007/s40592-023-00183-x.

## Introduction

It is a common refrain amongst phenomenologists, disability theorists, and feminist legal theorists that medical practice pays insufficient attention to people’s embodiment. Drew Leder, for instance, argues that traditional forms of physical examination which require the patient to adopt a ‘corpse-like pose, flat, passive, naked, and mute’^1^ fail to take adequate account of people’s first-person embodied perspective by focusing on disease and not the illness experience. The cause of this impersonal approach to care, Leder argues, is metaphysical: ‘the body is medically understood according to the model of a machine. The rest follows logically: need the mechanic speak lovingly to a car in the shop? Test it and fix it and give its owner the bill.’^2^ In a similar vein, S Kay Toombs argues that whereas ‘the physician sees the patient’s illness as a typical example of disease, the patient attends to the illness for its own sake.’^3^ The root cause of this, Toombs argues, is that physicians operate in a ‘purely scientific anatomical/pathological model of disease’^4^ which doesn’t incorporate an understanding of illness as a lived experience. More recently, Fredrik Sveneaus has argued that modern medicine has neglected the importance of phenomenological approaches to illness, resulting in an incomplete understanding of the person’s suffering.^5^

The complaint that we take insufficient account of people’s embodiment isn’t limited to the clinical interaction, it is also directed at health and welfare policy. Mark Flear, for example, argues that one of the reasons the harms caused by metal-on-metal hip implants and pelvic mesh implants have taken so long to come to official attention is due to the marginalisation of embodied experiences and first-person reports of pain within healthcare policy and the on-the-ground workings of the health care system. 6 The purpose of this paper is to consider both the arguments for *why* we ought to take people’s embodiment seriously, and *what* taking people’s embodiment seriously should look like in medical practice, health policy, and welfare policy. To do this, this paper brings feminist, phenomenological, and disability theory arguments regarding the importance of taking embodiment seriously into dialogue. Commonly, these are dealt with in somewhat of a siloed fashion within their own specialist sub-literatures. However, by bringing them together, this paper reveals that these arguments all pull in the same general direction. This, I suggest, opens up the possibility of finding a common solution to the concerns raised by feminist, phenomenological, and disability theory critiques.

To these ends, in section II I examine the argument that medical practice fails to take account of people’s embodiment, arguing that (where it does) this constitutes poor practice. The proper practice of medicine, I argue, must take account of people’s embodiment. The reason is that, unless medicine takes account of how people experience their embodiment, it is unlikely to achieve the goal of medicine, which, as I argue below, is healing. Although this might perhaps seem obvious, the work of phenomenologists, feminists, and disability theorists shows that this does not always occur in practice, where an overly a biomedical approach to the body can lead to people’s experience of their own embodiment being ignored or relegated to the side-lines.

In section III I then examine the claim that both health and welfare policy fail to take sufficient account of people’s embodiment, focusing specifically on the UK. Drawing on the results of the Cumberlege Review, as well as criticisms of the administration of both the Employment and Support Allowance (ESA) and Personal Independence Payment (PIP), I argue that, although public policy in general takes account of some features of people’s embodiment, the first-person perspectives of women and disabled people are not given the credibility they deserve. The result is the perpetuation of epistemic injustice.

Whilst the connection between not taking people’s differing embodiments seriously and epistemic justice has rightly been increasingly recognised in recent years,^7^ there has been relatively little discussion on some of the challenges to taking embodiment seriously in policy and practice. In section IV, I make a start at remedying this by setting up two not insubstantial challenges which have not hitherto been given the attention they deserve. The first is that there is an enormous amount of variation in how people are embodied. As a consequence, there is a strong possibility that adjusting policy to benefit particular individuals based on an appreciation of their embodied experiences could be detrimental towards other individuals. In short, given that taking people’s embodied experiences seriously might pull us in different, incompatible, directions; it is unlikely we will be able to satisfy everyone. Importantly, this is not to suggest that we should always aim to satisfy a majority of people. This is because the magnitude of the burdens imposed on a minority of individuals might be sufficiently large to outweigh smaller burdens experienced by a larger number of individuals. Instead, what I am suggesting is that it is unlikely that all policies we have reason to adopt as a response to certain individuals’ embodied experiences will be universally beneficial. If this is correct, we are forced to confront the question of how to allocate (and justify the allocation of) the benefits and burdens of these policies across the population.

The second challenge is how to ensure, in a public policy context, that decisions are fair, non-arbitrary, and based on serious deliberation about the facts of the matter. This is especially important given the (pernicious) pervasiveness and frequency with which the testimony of certain groups within the population is disregarded; for instance, that of women. However, whilst taking people’s first-hand accounts of their experiences into account is vital, it is equally important that people’s testimony is verified and subject to adequate scrutiny. This is particularly true when the outcome of a policy decision is the distribution of non-trivial benefits (such as state funding). The challenge is finding ways to ensure that people’s testimony is checked for reliability without this causing epistemic injustice. Having analysed these two challenges, I then move on, in section V, to argue that they can be (at least partially) overcome by adopting a just procedural approach to taking embodiment seriously and to make some novel suggestions for what a procedural approach to taking embodiment seriously should look like.

## Taking embodiment seriously in medical practice

### Failing to take embodiment seriously in medical practice?

The criticism that contemporary medicine pays insufficient attention to people’s first-person embodied perspective is common currency amongst phenomenologists, disability theorists, and in feminist theory. The task of this section is to unpack these arguments, starting with the claims made by phenomenologically inspired philosophers of medicine.

As we saw briefly above, phenomenologists such as Leder, Svenaeus, and Toombs, argue that medical practice is overly focused on identifying diseases, to the detriment of examining people’s experience of illness.^8^ This neglect of the first-person embodied experience of illness is often attributed to the underlying conception of the body as a machine which medical science is purportedly committed to.^9^ Focusing on disease leads to seeing sickness as merely bodily misfunction which can be corrected by medical treatment.^10^

Phenomenologically inspired philosophers of medicine argue that framing illness in this way has a number of consequences. First, as noted by Svenaeus^11^ and Havi Carel,^12^ medical practitioner’s focus on the notion of disease impedes proper communication in the doctor-patient interaction because it doesn’t respond to how the person in front of them conceives of the problem at hand. Whereas the clinician seeks to diagnose, treat, and offer a prognosis; the patient approaches the situation with a different set of values and needs, focusing on what illness means for them personally. They seek an explanation for their ailment, a cure which restores them to health, and a prediction of what will happen to them personally.^13^ This mismatch between what doctors and patients want can lead to patients feeling objectified in the clinical encounter.^14^ Svenaeus argues that this, in turn, can lead to the patient’s views not being taken fully into account when arriving at treatment decisions,^15^ making the patient ‘disappear from the attention of the doctor.’^16^ In Leder’s words:

If the patient is primarily a body-machine in need of repair, his/her personal interpretations, fears, wishes, and sufferings tend to become extraneous to the task at hand. Reductionist aspects of the paradigm lead to reductionist modes of treatment.^17^

Second, and relatedly, focusing on disease as opposed to the illness experience can lead to an increasing ‘layer of technological instrumentation designed to investigate the machine-body comes between physician and patient’,^18^ which can also lead to feelings of objectification.^19^

Third, Leder argues that the failure to focus on the illness experience means that insufficient attention is paid to how lifestyle, psychological, and environmental factors both cause disease and make it harder to cure, something which leads medicine to neglect both preventative measures and the psychological causes of bodily ailments.^20^ A fourth consequence of focusing on disease as bodily malfunction to the detriment of the illness experience is that ‘if a patient goes to the doctors with symptoms that the doctor cannot observe directly or verify independently of what the patient tells them […] the patient is liable to be told ‘there is nothing wrong with you’, regardless of how acute or debilitating his/her condition feels to the patient.’^21^

Analogous critiques can be found in the work of prominent disability theorists. For instance, Margrit Shildrick, Rosemary Garland Thomson, Susan Wendell, Elizabeth Barnes, and Deborah Kaplan all argue that medical practice focuses on the able body as the paradigmatic body, obscuring and marginalising disabled forms of embodiment in the process.^22^ Medicine, like wider society, operates on ableist assumptions that disability is not just difference, but negative difference.^23^ On this view, disabilities are generally understood as a harmed condition, as pathological, and/or as requiring correction.^24^ Medicine aims to both cure illness and disability, restoring people to normal, species-typical functioning; and prevent disabilities and illnesses from occurring if possible.^25^ In so doing, medicine plays in to the idea that the body can be controlled and changed to reduce disablement.^26^ This view, disability advocates argue, is inherently ableist, and the cause of ‘past abuses and present practices that cause people with disabilities to be isolated, overmedicated, and even killed as a means of solving the ‘problem’ of disability.’^27^

Instead of conceiving of disabilities as inherently bad, many disability theorists advocate a social model of disability according to which the badness of disability is primarily caused by society’s reactions to it.^28^ It is, therefore, unwarranted to assume that differences in embodiment are necessarily diminishing, or, by extension, that a person with an impairment is necessarily inferior.^29^ In fact, there are many ways of constructing a meaningful life with a disability,^30^ as most disabilities don’t preclude people from accessing meaningful goods such as happiness, rewarding relationships, personal achievements, and knowledge.^31^ Rather than seeking to normalise bodies, we ought to alter social systems that perpetuate oppression. In the context of medicine this will require not focusing exclusively on searching for a cure, we should also devote resources to enabling people to live well with their disabilities.^32^

The criticism that medicine fails to take account of people’s embodiment isn’t limited to phenomenologists and disability theorists, it can also be found in feminist bioethics and legal scholarship. Medicine, like wider culture, treats male bodies as the archetypical body.^33^ Male understandings of the body thus come to be taken as neutral and objective accounts of the body, as opposed to partial accounts of how some bodies are.^34^ This, in turn, leads to treating bodies that don’t conform (e.g. women’s bodies) as other, obscuring their embodiment in the process.^35^ This can be seen, for instance, in the fact that most medical and anatomical textbooks take the male body to be the ‘normal’ body, treating women’s bodies as deviations from the norm,^36^ sometimes relegated explicitly to a side box. As a result, we know less about female biology than male biology.^37^ Clinical trials also tend to underrepresent women,^38^ even when the conditions they study are more prevalent in women. Women are thus much more likely to suffer from medically unexplained symptoms than men.^39^ Although the underrepresentation of women in clinical trials has started to change in recent years, ‘many drugs were approved in the past without data on enough women to know whether they are equally effective in women and men.’^40^

This focus on the male body also leads to a comparative neglect of both (i) diseases and health conditions that mostly affect women, and (ii) how common diseases which affect both men and women manifest in women.^41^ As a consequence, women are more likely than men to be undertreated and misdiagnosed.^42^ Take heart disease as an example. As much of what is known about heart disease has been learnt from studying men, doctors are not as good as recognising the signs of heart attacks in women, where the symptoms can present differently. As a result, it can sometimes be misdiagnosed as anxiety, leading to worse outcomes.^43^

Women also experience more chronic pain conditions than men, including endometriosis, rheumatioid arthritis, and migraines. Chronic pain conditions, although they affect large numbers of people, attract less research funding than other conditions.^44^ Moreover, when women’s reports of pain are believed and responded to, women wait longer for pain relief^45^ and are generally given less pain relief than men.^46^ Women are also more likely to suffer from unexplained medical symptoms and contested conditions such as fibromyalgia, chronic fatigue syndrome, and chronic lyme disease.^47^ Often, prior to being diagnosed, women’s complaints are brushed off as the result of depression, anxiety, and/or stress.^48^ This failure to take the symptoms seriously, in turn, perpetuates our lack of knowledge surrounding these conditions, making them harder to understand and diagnose in the future.^49^

### Medicine as a healing practice

The arguments given by phenomenologists, disability scholars, and feminist theorists, and outlined above, point to the multitude of ways that the practice of medicine can fail to pay sufficient attention to how people are embodied. The fact that this still occurs in some real-world interactions with clinicians and other healthcare professionals is cause for concern. Given this, it is worth delving a little deeper into what the core problem is. Drawing on the work of some prominent philosophers of medicine, my argument, in this section, is that failing to taking embodiment seriously in the clinical interaction means that the goal of healing within medicine as a practice cannot be properly fulfilled.

Practices which aim at achieving particular values, such as medicine, can be said to have an internal morality which governs how those practices ought to be carried out. The list of practices with an internal morality is large and includes most ‘liberal professions’ such as journalism, accountancy, scientific research, engineering, legal practice, architecture, urban design, teaching, and medicine. In this paper, the focus will be on how the internal morality of medicine requires taking embodiment seriously, leaving open the possibility that analogous arguments could be made for other practices.

One popular account of the purpose of medicine is that medicine aims at healing.^50^ For philosophers of medicine such as Edmund Pellegrino, Richard Baron and Drew Leder, medicine is centred around a therapeutic relationship between the person with an affliction and the professional who professes to heal.^51^ Healing, on this account, is not just about the treatment of diseases, it is also about the alleviation of suffering caused by the illness experience.^52^ As suffering is something that can only be experienced first-hand, the patient has expertise regarding their suffering.^53^ In order to effectively alleviate this suffering, medical professionals need to understand it.^54^ Given that only the person suffering can tell us what their suffering is like,^55^ patients are in a better epistemic position to identify unmet needs and the burdens of illness management than clinicians.^56^

Alleviating suffering, then, will likely require inquiring into how people experience their embodiment, leading to more patient-focused doctor-patient interactions.^57^ As patients are required to provide their expertise about how they experience their illness, focusing on embodiment requires that patients take a more active role in their treatment. Leder, for example, argues that taking embodiment seriously is incompatible with traditional models of the physical examination in which ‘the patient is asked to assume a corpse-like pose, flat, passive, naked, mute.’^58^

Taking patients self-reports seriously can also be justified on the basis of avoiding misdiagnosis and ensuring people receive proper care.^59^ People’s self-reports have evidential value for clinicians attempting to diagnose a person’s ailment. They do so both by providing evidence for hypothesis generation and by helping us rule out certain hypothesis.^60^ As a consequence, ignoring or dismissing patient self-reports can lead to misdiagnosis, delays in diagnosis,^61^ or inadequate treatment.

The importance of inquiring into people’s first-hand experiences of using medical technology to ensure proper care can also be illustrated by considering the use of pacemakers. Pacemakers are ‘small, battery powered generators that supply electric pulses to the heart when the heartbeat is too slow.’^62^ Standard pacemakers consist of two main components: a battery powered pulse generator that emits electric pulses, and one or more leads that are inserted into the heart muscle to deliver the electric stimulation. Individuals who receive a pacemaker attend ‘pacing clinics’ to find the appropriate level of stimulation (i.e. the frequency of the pulses emitted by the device).^63^ In these clinical interventions people’s heartrate is sped up and slowed down while patients are asked about how they feel. Set the pacemaker too high and people feel agitated. Set the pacemaker too low, and people can feel tired. The goal is to find a level of stimulation that protects against the risk of heart failure whilst remaining comfortable for the user.

A similar process is required for cochlear implants. Cochlear implants are electronic devices that enable deaf people to receive auditory sensations. Cochlear implants are made up of an external device and an internal implant. The external component contains a microphone, speech processor, and transmitter which is attached to the skull behind the ear. The internal implanted component consists of a receiver and an electrode array which is surgically implanted into the cochlea. Cochlear implants work by capturing sounds from the environment, converting them into electrical signals, and using these signals to directly stimulate auditory nerves in the cochlear, bypassing non-functioning parts of people’s ears.^64^ Cochlear implant recipients attend ‘mapping sessions’ in which a technician fine tunes the voltage the electrodes deliver to adapt the device to the needs of the recipient.^65^ The goal is to help recipients ‘re-learn how to hear’, taming the initial ‘chaos of sounds’ and turning them into ‘identifiable, meaningful patterns’.^66^ Unless this is done properly, the experience can be unpleasant, and the device won’t fulfil its purpose of enabling people to identify sound.^67^ In both of these cases, setting the device to ensure it works for the patient involves inquiring into their experience of their embodiment and adjusting device functioning in light of this information.

Taking embodiment seriously in this way can be seen as a way of avoiding epistemic injustice. Epistemic injustice is injustice done to someone specifically in their capacity as a knower.^68^ There are two main types of epistemic injustice: hermeneutic injustice and testimonial injustice. Hermeneutic injustice occurs when ‘a gap in collective interpretative resources puts someone at an unfair disadvantage when it comes to making sense of their social experiences’.^69^ Testimonial injustice occurs ‘when prejudice causes a hearer to assign a deflated level of credibility to a speaker’s testimony’.^70^ Testimonial injustice is prevalent in healthcare,^71^ as ill people are subject to prejudicial stereotypes that tend to deflate their credibility.^72^ In the words of Ian Kidd and Havi Carel, ill people are conceived of as:

cognitively impaired or emotionally compromised, owing either to their somatic condition or their psychological reactions to it […]; or as existentially unstable, gripped by anxieties about mortality and morbidity such that they ‘cannot think straight’; or that they will be psychologically dominated by their illness in a way that warps their capacity to accurately describe and report their experiences (e.g. ‘the moaner’ or ‘the drama queen’ stereotype).^73^

The problem is compounded for individuals who have other characteristics which are also associated with prejudicial stereotypes.^74^ Women, for example, often report disbelief from physicians,^75^ with many of the women interviewed during the Cumberlege Review reporting struggling to be heard or believed by clinicians.^76^ Some interviewees described having their painful symptoms described ‘as ‘normal’ and attributable to ‘women’s problems.’^77^

Inquiring into people’s embodiment is also important because how we are embodied shapes our perception of what is valuable and meaningful in life.^78^ If the purpose of clinical interactions is to assist people in making medical decisions that are right for them, inquiring into people’s first-person perspectives might help clinicians support patient decision-making.^79^ Whether a particular medical intervention is right for a particular person will depend on their values and situation. Side-effects that might be tolerable for some might be intolerable for others and vice-versa. Understanding more about a person’s values, their lived experience, and what gives them meaning in life will be valuable information in deciding the best course of action for the patient in the clinical consultation, shifting the onus away from clinical measures of medical success.

As well as being important in the clinical interaction, taking people’s experience of their embodiment seriously has wider epistemic benefits. First, it could also lead to improvements to medical practice or the design of medical devices more generally.^80^ As Flear argues, the current knowledge base for regulation is primarily based on scientific-technical knowledge. The result is a blind spot when it comes to patient understandings of harm.^81^ As patients are in a better epistemic position to identify unmet needs, inquiring into people’s experiences of, for instance, using a medical device will reveal sources of frustration, sticking points, and avenues for improvement.^82^ These insights can then be used by manufacturers in their development of mass produced products.^83^ They can also be used by embodied health movements and patient groups to influence policy and lobby for better services.^84^ Embodied health movements are a type of social movement focusing on disease, disability, and/or illness experience which aim to challenge existing medical knowledge and practice to ensure better treatment and prevention of particular health conditions.^85^ These movements often aim to establish collaborative relationships between patients and researchers with a view to gaining ‘a place at the scientific table so that their personal experiences can help shape research design’.^86^

A good example of an embodied health movement is the #WeAreNotWaiting movement. Tired of waiting for commercial companies to produce effective technological solutions that fully meet their needs, some people with diabetes (sometimes known as ‘loopers’) took it upon themselves to develop DIY Automated Insulin Delivery Systems or so-called Artificial Pancreas Systems.^87^ Frustrated by the lack of interoperability between insulin pumps (which deliver insulin) and continuous glucose monitors (which measure blood sugar levels), a community of users developed their own solution: an algorithm hosted on a small computer or a smartphone which connects the two devices together and partly automates insulin delivery. There are a number of different open-source systems (e.g. Loop, OpenAPS, and Android APS) all of which work on similar principles.^88^ Since their inception in the type 1 diabetes community, major medical device manufacturers such as Medtronic (Minimed 780G)^89^ and Tandem (t:slim X2 with control-iQ technology)^90^ have developed their own commercially available automated insulin delivery systems. Whilst there is not the space to go into the detail of this community-led movement here,^91^ what is significant is that the diabetes community has - via their own innovations and initiatives - managed to raise the visibility of the unmet needs of people living with diabetes and the potential of DIY solutions.

This example shows that taking users’ embodied experiences seriously is important for the development of medical devices because, if the sticking points they are uniquely placed to identify go unresolved, people who might benefit from using a particular medical device or medicine will not use them,^92^ thus undermining the point of developing them which is, presumably, to benefit people.

To sum up, phenomenologists, disability theorists, and feminist philosophers converge on the claim that medical practice fails to take people’s embodiment seriously. Phenomenologists argue this is due to the fact medicine focuses too much on disease, ignoring patients’ first-person perspectives on illness. Disability theorists argue medicine fails to take the embodied reality of disability seriously by conceiving of disability as a form of negative difference in need of correction, thus obscuring the fact that for many disabled people, disability is not inherently negative. Feminist theorists argue that medical practice has hitherto focused almost exclusively on male anatomy, which leads to the disregarding of illnesses that primarily affect women. Building on these insights, what I have argued here is that, when medicine fails in these ways, it cannot fulfil its goal of healing. Healing isn’t simply a matter of curing disease, it also requires the alleviation of suffering. Unless medicine takes embodiment seriously, it runs the risk of: (i) misdiagnosing people’s ailments (thus delaying care), (ii) perpetuating epistemic injustice, and (iii) recommending treatment regimes which do not align with people’s values and/or actual needs.

## Taking embodiment seriously in health and welfare policy

Medical practice isn’t the only legitimate target of the kind of critiques I have been considering in this paper. They can also rightly be directed at UK health and welfare policy more generally. In other words, it isn’t just that some individual practitioners fail to take account of people’s embodiment in the clinical interaction, the problem, it is argued, is systemic.

Failure to take embodiment seriously in health and welfare policy isn’t merely of academic concern, it can also be the cause of real-world harm.^93^ The recent Independent Medicines and Medical Devices Safety Review, led by Baroness Cumberlege, demonstrates the magnitude of these harms. Baroness Cumberlege was tasked with examining ‘how the healthcare system in England responds to reports about harmful side-effects from medicines and medical devices.’^94^ One of the treatments the review focused on was pelvic mesh implants, which are used in the surgical repair of pelvic organ prolapse and the management of stress urinary incontinence.^95^ As a result of having pelvic mesh implants, many women suffered serious harmful side-effects including chronic pain, nerve damage, organ and tissue damage, and limited mobility.^96^ Importantly for the purposes of this paper, the review found that failure to take adequate account of these concerns contributed to the serious side-effects of pelvic mesh implants being ignored.^97^ In the words of the report:

Women, in reporting to us their extensive mesh complications, have spoken of excruciating chronic pain feeling like razors inside their body, damage to organs, the loss of mobility and sex life and depression and suicidal thoughts. Some clinicians’ reactions ranged from ‘it’s all in your head’ to ‘these are women’s issues’ or ‘it’s that time of life’ wherein anything and everything women suffer is perceived as a natural precursor to, part of, or a post-symptomatic phase of, the menopause.^98^

Had these women’s perspectives been taken more seriously, the amount of people suffering these harms could potentially have been reduced. The problems uncovered by the Cumberlege Review, however, are not unique.

More recently, the Ockenden review also found that failures to listen to women’s descriptions of their illness symptoms and complaints about poor service contributed to avoidable harm to mothers and babies in the UK.^99^ In her review of the failings at the maternity service at Shrewsbury and Telford NHS Trust between 2000 and 2019, Donna Ockenden found cases where women’s pain was downplayed,^100^ symptoms indicative of rectovaginal fistulas ignored,^101^ and reports of tightening and reduced foetal movement were not acted upon quickly enough,^102^ all of which contributed to poor outcomes for mothers and babies. Moreover, when families raised concerns about their care with the Trust, investigations were handled inadequately and not escalated. As a consequence, problems with care were not identified, leading to missed opportunities to learn from mistakes and improve the service provided by the trust.^103^

Another example of health policy failing to take some people’s first-person perspective on their embodiment seriously is the fact that, when determining Quality Adjusted Life Years (QALYs), researchers determine the utility of a given health state by asking a representative sample of the population about their preferences for particular health states.^104^ The reason we should be suspicious of this methodological choice is due to the existence of the disability paradox. The disability paradox is the term given to the mismatch between disabled people’s self-assessment of their quality of life (which can be high) and the assessment made by non-disabled people (generally lower).^105^ The disability paradox powerfully illustrates how difficult it can be to estimate how other people experience their embodiment from afar.^106^ If we really want to know if/how people’s quality of life is affected by having a certain medical condition, we ought to ask people living with that condition.^107^

Failing to take people’s embodiment seriously is also prevalent in the implementation of welfare policy in the UK. These failures can be seen in both the administration of the UK government’s Employment and Support Allowance (ESA) and Personal Independence Payments (PIP). The purpose of ESA is to provide an income to people who have become too unwell to work. In order to claim ESA, people must first get a medical certificate issued by their GP. Once received by the UK’s Department for Work and Pensions (DWP), they enter an assessment phase during which their eligibility is assessed. Claimants must then fill in a Limited Capability for Work Questionnaire. In some instances, eligibility can be determined on the basis of the paperwork alone. However, most claimants will require a face-to-face assessment, known as a Work Capability Assessment (WCA) to determine whether they are fit for work and, consequently, the level of benefits they are entitled to.^108^ The assessment is conducted by a healthcare professional contracted by outsourcing companies such as Atos and Capita. During the assessment, a person’s disability is assessed against a series of 17 functional descriptors such as: ‘cannot unaided by another person mount or descend two steps even with the support of a handrail’ or ‘cannot raise either arm to top of head as if to put on a hat’.^109^ Each descriptor is given a number of points. In the examples above, being unable to mount or descend two stairs carries 9 points and being able to raise either arm to the top of one’s head carries 15 points. To be granted benefits, a person needs to score 15 points in total across all descriptors.^110^

The WCA has been widely criticised since its implementation in 2008.^111^ There have been widespread reports from claimants that assessors did not seem to believe their testimony, did not document their answers to questions asked in the assessment accurately,^112^ did not document crucial information,^113^ and made unjustified extrapolations from claimant’s statements or their behaviours.^114^ For instance, if a claimant makes it to the assessment centre, that is taken as evidence that they are able to travel, walk, sit comfortably, and cope with social interactions (which is not necessarily true). If follow-up questions are not asked, the assessor may get an erroneous impression of whether the person is able to do the prescribed activity without pain or discomfort, for example.

One way of understanding these complaints is as instances of epistemic injustice. In instances where claimants are simply not believed, people are being subjected to testimonial injustice in that their testimony is given deflated level of credibility in virtue of them being disabled. In cases involving unjustified extrapolations from statements and lack of follow-up on ambiguous statements, the epistemic injustice seems to involve a lack of due care and concern in getting what one is saying right. The problem isn’t strictly that their testimony is not being believed, it is more that their statements are being interpreted uncharitably.

Similar complaints have been raised against Personal Independence Payment assessments. PIP is a non-contributory, non-means-tested benefit which aims to compensate people with disabilities for the extra costs involved in living with a disability.^115^ As with ESA, entitlement to PIP is not based exclusively on one’s diagnosis. Instead, it is assessed on the basis of functional criteria which aim to determine how much support people need on a day-to-day basis. As with Work Capability Assessments, there have been a number of problems with the administration of PIP assessments. Claimants report being disbelieved,^116^ inaccurate information being included in reports,^117^ and unjustified extrapolations from statements or behaviour.^118^ Peter Allridge, for instance, found that his PIP assessor had determined that he was able to walk 50 m based on the unrelated fact that he was able to drive a car with unmodified pedals.^119^

In sum, the problem of people’s embodiment not being taken seriously is not limited to individual clinical interactions, is also prevalent in the UK at a policy level, causing real-world harm. It can be seen in the failures uncovered by the Cumberlege and Ockenden reviews, the continued use of QALY’s to assess the efficiency of medical interventions, and the epistemic injustices suffered by benefits claimants.

## Challenges to taking embodiment seriously in public policy

So far we have seen how public policy can fail to take people’s embodiment seriously. However, even if we agree that we ought to take embodiment seriously, there are still challenges to doing so in practice.

The first challenge is that different people are embodied differently. Consequently, it will be difficult (if not impossible) to craft policy that satisfies everyone. The second challenge concerns how to ensure that, when we subject people’s reports of their first-person embodied experiences to critical scrutiny, we don’t end up perpetuating epistemic injustice. This section thus sets the stage for section V, where I suggest that a procedural approach can help us overcome these challenges. With this brief outline of what is to come in mind, let us turn to the first challenge to taking embodiment seriously: the enormous amount of variation in our embodiments.

Different people are not only differently embodied, but also differentially experience similar embodiments. First, the experience of illness, for example, will depend on the individual. This can be seen most clearly when it comes to pain reports, which are only measurable using subjective scales and metaphorical descriptions of the sensations (e.g. shooting, stabbing, or throbbing pains). Second, what would alleviate suffering for people will also likely depend on the individual’s experience. This presents a challenge for integrating substantive embodiment claims into public policy. The reason is that, given this variation, there might be scenarios in which doing what is required to alleviate the suffering for one group of users might come into conflict with doing what is required to alleviate the suffering of others.^120^ Moreover, given that all of these accommodations have costs, and we exist under conditions of moderate scarcity, choices about which issues to respond to first will have to be made.^121^

Tom Shakespeare gives a helpful example of this tension. Wheelchair users (and other groups of people including pram pushers) benefit from forms of level access to buildings and public spaces such as ramps and kerb cuts. However, kerb cuts can make it harder for blind individuals to distinguish pavement from road, potentially exposing them to danger. On the flip side, wheelchair users (or pram pushers) can find the tactile paving used to indicate hazards (such as zebra crossings or platform edges) difficult to navigate.^122^ To give another example: bright lighting and strong colours can help visually impaired people navigate the built environment. However, for people who are hypersensitive to visual stimuli, as some people with autism are, these stimuli can prove overwhelming, making that environment less accessible to them.^123^

Finally, consider cochlear implants. Some members of the Deaf community are opposed to cochlear implants on the grounds that they make it harder to sustain an important element of Deaf culture: sign-language.^124^ Other people see cochlear implants as beneficial forms of technology that benefit them by making communication easier in a world in which sign-language is marginalised.^125^ Whatever policy we adopt on cochlear implantation, the policy will impose burdens on some people. If we adopt a policy of promoting cochlear implantation, sustaining Deaf culture might be harder if such a policy means that there are fewer potential members of the Deaf community, with a concomitant reduction in numbers of those who know sign language and who feel culturally part of the community.^126^ If we prohibit or otherwise aim to reduce cochlear implantation (e.g. by not including it on the NHS), individuals who could have benefited from a Cochlear implant and would have wanted one will not receive one.^127^

The problem, however, is not limited to tensions between people with different impairments. Shakespeare argues that:

people with the same impairment may require different accommodation, both because everyone experiences their own impairment differently, and because each impairment comes in different forms, and because different people have different preferences for solving impairment problems.^128^

In some instances, these tensions might be resolvable. Perhaps the current incompatibility is based on a lack of imagination in finding solutions. Perhaps some enterprising urban designer can devise a new form of road design which both allows us to both do away with kerbs whilst still allowing blind individuals to distinguish pavement from road. In the case of cochlear implants, we could mitigate the risks they pose to Deaf culture by only allowing cochlear implants in parallel with a policy aimed at promoting the acquisition and use of sign language among both hearing and non-hearing people.^129^ What we should not do, however, is assume that all tensions such as these can be resolved if only we could sufficiently engage our collective imagination.^130^ It may turn out that in some cases no middle ground is possible. As such, if we are going to integrate claims about people’s embodiment into public policy, we need to have some way of resolving situations in which we are pulled in mutually incompatible directions and no middle ground exists.

The second challenge concerns how to ensure that people’s testimony about their first-person embodied experience is subject to adequate scrutiny without this resulting in epistemic injustice. As we have seen, some groups of people’s testimony is given a deflated level of credibility in virtue of prejudiced stereotypes about their identity. At first sight it might seem like the solution to this is simple: believe what people say. However, in public policy contexts we need to be able to interrogate people’s testimony, which precludes that we simply take people’s testimony at face value.

There are three reasons for this requirement. The first reason is that, when making assertions in public policy contexts, participants make an implicit commitment that what we are saying is true and supported by reasons. This, in turn, creates a commitment to justify the claims we make to others by supplying the reasons should we be asked to do so.^131^ Part of being a reasonable participant in a public policy context is being open to this call for justification by demonstrating a ‘willingness to listen to others who want to explain to them why their ideas are incorrect.’^132^

The second reason is that subjecting testimony to adequate scrutiny is necessary to ensure that a decision-making procedure is non-arbitrary. One way in which public policy decisions can be arbitrary is by not being based on facts.^133^ Thus, to ensure non-arbitrariness in public policy contexts, we need to inquire into the validity of any testimony used as evidence in deliberations about public policy. Unless we do so, it will be difficult to justify the view that decisions are non-arbitrary.

The third, related reason, for subjecting people’s first-person accounts of their embodied experiences to scrutiny is connected to the idea that people’s embodiment can determine the level of state support they are entitled to and the extent of their obligations to contribute to the cooperative scheme. As Shakespeare puts the point:

it is unfeasible that someone could be entitled to public services simply because they decide they want them. A process is needed to legitimate and allocate support in a welfare state. Medical diagnosis and welfare assessments are the prime means by which an individual gets a disabled parking badge, a welfare benefit to cover the extra cost of a condition, or access to a disability pension if they are unable to work.^134^

Given that the benefits being distributed are non-trivial, we have reason as a matter of fairness to ensure that they are delivered to those who are entitled to them and not delivered to those who are not.^135^

The challenge, however, is doing this in a way that doesn’t perpetuate testimonial injustice. As we saw above, testimonial injustice occurs when ‘prejudice causes a hearer to assign a deflated level of credibility to a speaker’s word’.^136^ The questions we need to answer are: What forms of critical scrutiny are compatible with the demands of epistemic justice? Can we scrutinise the validity of people’s testimony without allowing prejudice to taint our estimation of a person’s reliability? How can we minimise the risk of testimonial injustice in the process of scrutinising people’s claims?

In sum, there are two challenges to taking people’s embodiment seriously in public policy contexts. The first is ensuring that, where a change is not universally beneficial, we have a fair procedure for allocating the benefits and burdens of such a change. The second is finding a way of ensuring that people’s testimony can be scrutinised without this resulting in epistemic injustice. It is to these questions we now turn.

## A procedural approach to taking embodiment seriously

It seems that the solution to both these challenges is to develop some form of just procedure for both soliciting testimony about people’s embodied experiences and taking it adequately into account in the policy development process. The reason we need to adopt a procedural approach is that, given the diversity of people’s embodiment, whatever policy we end up favouring will have disparate effects on different groups, creating different patterns of advantage and disadvantage. As we can’t simply adapt policy in a universally beneficial way, the demand that people’s embodiment be taken seriously should be interpreted as requiring that people’s embodied experience be *adequately considered* in the process of deciding what the policy should be. Given this, any just procedure will need to confront two sets of questions. The first set of questions concern who should be entitled to participate in the policy development process: Should participation be limited to those with professional expertise, or should participation be extended to patient advocacy groups, patients themselves, or the public at large?

Taking people’s embodiment seriously requires including patient advocacy groups and affected persons as well as those with professional expertise. The reason being that it is difficult to see how we could take people’s perspective into account without providing for some way of people making their voices heard.^137^ In this sense, inclusion in the process is a precondition of taking a person’s embodiment seriously. Professional expertise, whilst important, cannot substitute for direct participation. The reason being that patients, service users, and members of the public have insight that professional experts do not necessarily have access to.^138^ Whereas a clinician, for instance, has expert knowledge on some aspects of health, patients themselves have knowledge of their own lives, circumstances, and experiences; all of which are important to evaluating the quality of healthcare services.^139^ Often service users also have a unique understanding of the realities of service delivery on the ground and of effective self-management techniques for their conditions.^140^ In other words, they have experiential knowledge, that is ‘truth learned from personal experience with a phenomenon rather than truth acquired by discursive reasoning, observation, or reflection on information provided by others.’^141^

The disability rights movement provides a good illustration of the importance of inclusive participation. In his book *Nothing About Us Without Us*, James Charlton argues that campaigns organised by disabled people for disabled people have been instrumental in achieving change on a range of issues including:

the inaccessibility of public transportation; the lack of accessible, affordable housing, the institutionalizing of poor, young people with severe disabilities in nursing homes because of the prohibitive cost of personal assistance.^142^

Given that not everyone who is affected by a decision can participate in the making of it, decision-making bodies will need to make use of the notion of representation. There are numerous forms this representation can take, including public consultations, participatory needs assessments (which bring together professionals and service users to identify priorities),^143^ the inclusion of patients on the boards of advocacy organisations or as participants in institutional committees,^144^ the inclusion of advocacy organisations in policy discussions,^145^ reserved seats for patients or their representatives in policy-making fora,^146^ service user audits (which would empower users of services to monitor the delivery of services they use),^147^ or citizen juries and assemblies (in which representative samples of citizens meet to deliberate about specific issues).^148^ Which form of participation is best will depend on the situation. For the purposes of this paper the important points are that a just procedure will require inclusive participation and there are numerous ways in which opportunities for participation might be increased.

In order for this participation to be meaningful, however, decision-making bodies that include lay participants and those affected by particular conditions have to have some form of influence on public policy.^149^ If the participatory bodies are merely consultative and have no influence on those who make decisions, they merely pay lip service to the idea of taking people’s embodied first-person perspectives seriously.^150^ In order for participatory mechanisms to fulfil their role, they must have some influence on the policy process. In other words, decision-makers need to be accountable to these participatory bodies.

Once we agree that just procedure will need to be inclusive, a second set of issues arise: i.e. those concerning how the experiences and views of those participating in the policy proposal ought to be considered within the policy process. These include: How should patient testimony be solicited? Should patient testimony be questioned and/or interrogated during the process? If so, how can this be done in an epistemically just way? To answer these questions, we need to delve deeper into what avoiding epistemic injustice requires. On Miranda Fricker’s account of epistemic injustice, avoiding epistemic injustice requires we adopt a stance of ‘critical openness to the word of others,’^151^ correcting for the influence which identity prejudices could have over a person’s initial credibility assessment.^152^ The goal is ‘to neutralise any negative impact of prejudice in one’s credibility judgments by compensating upwards.’^153^ This, however, does not bar us from sometimes attributing a credibility deficit to some people’s testimonies. On Fricker’s account, testimonial injustice occurs when testimony is devalued because of *prejudice* against a person’s social identity.^154^ If credibility deficits are attributed to a person’s testimony for non-prejudiced reasons, no epistemic injustice need occur. To illustrate this, let us return to the example of people’s testimony about their pain not being believed.

As mentioned above, women often have their testimony about pain devalued in clinical encounters based on prejudiced views about women overreacting or being overly sensitive to pain. This is a paradigmatic case of testimonial injustice. This, however, does not mean we have to take a person’s testimony about pain at face value in all circumstances. In some cases, there may be good (non-prejudiced) reasons for not taking a person’s self-report of pain at face value. Jackie Leach Scully and Catriona MacKenzie, for example, suggest that:

A willingness to be open to, respectful, and sympathetic towards, the perspective of another does not mean that we cannot engage in critical assessment of the other’s views or check the accuracy of her testimony. For people’s representations of their experience can be compromised by self-deception, manipulation by others, or self-interested motives, and constrained by lack of resources and opportunities, insufficient information, restricted horizons, and so on.^155^

Creating and implementing a just procedure will require training people to avoid epistemic injustice in their dealings with people. The goal is to ensure that, when inquiring into a person’s first-person perspective, people are treated as epistemic peers^156^ and their testimony is given due consideration. 157 The question then is, how can this be done? The literature on epistemic injustice provides us with some suggestions, most of which focus on providing enhanced training for people who are tasked with soliciting testimony.

Carel and Kidd, for example, argue that epistemic injustice can be reduced by training both those who solicit and those who give testimony to use a phenomenological toolkit.^158^ The phenomenological toolkit aims to promote epistemic symmetry^159^ between the parties by providing

ill persons with the means of overcoming the inarticulacy and ineffability of their experience of illness and healthcare practitioners with the hermeneutical resources required for better understanding those testimonies.^160^

It does so by guiding people through a structured thought process consisting of three steps during a one-day workshop. The first step in the method consists in assisting patients to focus their attention on their illness experience as it appears to them, instead of on the disease that is causing the illness experience.^161^ The second step involves ‘thematizing’ one’s illness, i.e. attending to the phenomenon in such a way as to make particular features of it explicit.^162^ The goal of this exercise is to understand the multidimensional nature of illness, helping us see different people’s experiences of illness within their wider context. The third step is to consider how illness ‘changes one’s being in the world’.^163^ The goal is to

capture the pervasive effects illness may have on one’s sense of place, on one’s interactions with the environment and with other people, on meaning and norms, and on the nexus of entities, habits, knowledge, and other people that makes up one’s world.^164^

This all seems like a step in the right direction. Nevertheless, resources such as the phenomenological toolkit will only take us so far. They won’t reduce epistemic injustice on their own. Such resources can help us perceive others sympathetically,^165^ thereby avoiding devaluing other people’s reports of their own experience, only if the types of reflection they aim to stimulate become habitual. In this sense, the ability to perceive sympathetically, facilitate inclusive and equitable deliberative exchanges, and avoid epistemic injustice in our dealings with others are like virtues.^166^ Virtues must be trained and developed through reflexive praxis guided by more experienced practitioners.^167^ Acquiring a virtue isn’t simply a matter of following rules. It also involves developing the right attitudes and skills. Ensuring a procedure for gathering testimony is just will thus require training and guidance over time. One way in which this can be achieved is by having novices shadow more experienced practitioners until the skill of the mentor is acquired by the novice.^168^

Testimonial injustice, however, is only part of the problem. A second problem is caused by hermeneutic injustice, i.e. the lack of shared cultural resources with which to express one’s lived experience. Reducing testimonial injustice is likely to require reducing hermeneutic injustice. The reason for this is that, unless people have the cultural resources with which to make sense of their cultural experiences, they will struggle to make themselves intelligible in testimonial exchanges. As a consequence, they are more likely to have their testimony devalued.^169^

Mitigating hermeneutic injustice will require both individual and collective changes. On a collective level, reducing hermeneutic injustice will require reducing hermeneutic marginalisation,^170^ that is: redressing the imbalance of power that generates skewed epistemic resources. In practical terms, this could be achieved by providing and fostering spaces for people to develop their own terminology to account for their experience.^171^ Embodied health movements and increased stakeholder participation may be particularly helpful in this regard, as would widespread usage of tools such as the phenomenological toolkit.^172^

On an individual level, mitigating hermeneutic injustice will require adopting an attitude of ‘alertness or sensitivity to the possibility that the difficulty one’s interlocutor is having as she tries to render something communicatively intelligible is due not to its being nonsense or her being a fool, but rather to some sort of gap in collective hermeneutical resources’.^173^ The goal is to seek to actively seek to counteract the impacts of hermeneutical marginalisation by being reflexively aware of one’s social identity may hinder one’s own understanding,^174^ and interrogating the boundaries of one’s interpretative horizons.^175^ Jose Medina suggests a number of ways in which this alertness can be given expression in conversation, including ‘knowing when to shut up, knowing when to suspend one’s own judgement of intelligibility, calling critical attention to one’s own limited expressive habits’,^176^ checking whether other differently situated people find it intelligible, and letting other people set the tone and pace of the interaction.^177^

To sum up, the idea that we ought to take embodiment seriously shouldn’t be interpreted as requiring that we directly adjust policy to accommodate people’s embodiment. It is best interpreted as a procedural demand. I have argued that any just procedure will have to confront two types of questions. The first set of questions concern who should be included in the procedure. The second set of questions concern how the procedure should solicit and evaluate people’s embodied experiences. With regards to the first set of questions, I have suggested that a just procedure that takes people’s embodiment seriously will be inclusive. With regard to the second set of questions, I have argued that taking embodiment seriously will require soliciting people’s testimony and questioning it in a way consistent with avoiding epistemic injustice.

## Conclusion

The idea that medicine and health and welfare policy fail to take people’s embodiment seriously has rightly been highlighted and critiqued in a number of phenomenologically-inspired literatures, including in the philosophy of medicine, feminist scholarship, and disability theory. In this paper, I have sought to provide an interpretation of the demand that embodiment be taken seriously in these realms, and to consider how this could be achieved.

To begin, I argued that, although medical practice has failed to take people’s embodiment seriously, doing so is inconsistent with the proper practice of medicine. Drawing on Edmund Pellegrino’s account of the purpose of medicine, we saw that, if medicine is aimed at healing, there are a number of reasons why it must take people’s first-person embodied perspectives seriously. First, healing involves the alleviation of suffering. Given that suffering is experienced first-hand, alleviating suffering will require inquiring into a patient’s first-person perspective of what it is like. Second, taking people’s self-reports seriously is important if medicine is to ensure the correct diagnosis of disease. People’s self-reported symptoms can provide evidence for hypothesis generation, and help to rule out hypothesis of what explains people’s illness symptoms. Third, taking people’s first-person embodied perspectives seriously is also important in deciding which form of treatment is best. Different people value different things and tolerate different side-effects differently. Hence, inquiring into people’s embodiment will provide valuable information for advising people on the best course of treatment.

These three reasons for taking embodiment seriously have implications for the proper practice of medicine. Notwithstanding, the acute time and other resource constraints which bedevil medical practice, healthcare professionals who take their obligation to heal seriously (which arguably they ought to) would be well-advised to inquire into patient’s lives, listen sympathetically to their self-reports, and work to mitigate the impacts of prejudicial stereotypes on their evaluation of people’s testimony, if they want to effectively diagnose and treat people’s ailments. Achieving these goals, though, is not simply a matter of the behavious of individual clinicians, it is also a matter of healthcare policy. All of these activities take time and training. When both are in short supply, there is a limit to what can be achieved by individual clinicians. If we care about healing therefore, we ought to support a well-resourced healthcare system in which clinicians have time to inquire into people’s first-person embodied experience.

Where the (infra)structure of the healthcare system does not adequately do this, the consequences can be grave (sometimes even fatal). This we saw in the Cumberlege and Ockenden Reviews, both of which demonstrated how ignoring patient’s first-person perspectives can lead to avoidable harms. Likewise, similar issues abound in other policy areas. To illustrate this, I drew on two examples from social welfare policy (although it need not be limited to these). I showed that claimants of ESA and PIP report being disbelieved and having their testimony misrepresented during assessments, leading to denial of benefits to which they were entitled. There are thus serious real-world consequences where people’s embodiments are in essence epistemic blind spots in the making and implementation of public policy.

These examples show that we ought to take people’s embodiment more seriously in the crafting and implementation of public policy. The question is: how? In answering this, I outlined two challenges to taking people’s embodiment seriously which need to be overcome. The first is that there is an enormous amount of variation in how people are embodied. The second is that we need to find a way to balance subjecting people’s testimony to adequate scrutiny and avoiding epistemic injustice. My suggestion is that the solution to both these challenges is the creation of an inclusive participatory procedure for determining policy in which participants are encouraged to deliberate in an epistemically just way. Whilst, in this paper I have left open the question of precisely what form this procedure should take, if public policy professionals are to meet their obligations to the public (to craft fair and non-arbitrary public policy), they would do well to give people’s first-person perspectives on their embodiment greater influence in the policy development process.

### Electronic supplementary material

Below is the link to the electronic supplementary material.

Supplementary Material 1

## Footnotes

1 Leder, Drew (1992) ‘A Tale of Two Bodies: The Cartesian Corpse and the Lived Body’ in Leder, Drew (ed) *The Body in Medical Thought and Practice*, Springer. p. 22.

2 Leder, Drew. (2002) ‘Whose Body? What Body? The Metaphysics of Organ Transplantation’ in Cherry, Mark (Ed) *Persons and Their Bodies: Rights, Responsibilities, Relationships*. Kluwer Academic Publishers, New York. p. 240.

3 Toombs, S. Kay. (1987) ‘The Meaning of Illness: A Phenomenological Approach to the Patient-Physician Relationship’ *The Journal of Medicine and Philosophy*, Vol. 12, pp. 229.

4 Ibid, p. 221.

5 Svenaeus, Fredrik. (2018) Phenomenological Bioethics: Medical Technologies, Human Suffering, and the Meaning of Being Alive. Routledge, London p. 42.

6 Flear, Mark. (2019) ‘Epistemic Injustice as a Basis for Failure? Health Research Regulation, Technological risk and the Foundations of Harm and Its Prevention’ *European Journal of Risk Regulation*, Vol. 10, No. 4, p. 707–709.

7 Carel, Havi. (2014) ‘Epistemic Injustice in Healthcare: A Philosophical Analysis’ *Medicine, Healthcare, and Philosoph*y, Vol. 17, p. 532; Carel, Havi. (2016) Phenomenology of Illness. *Oxford University Press*, Oxford. p. 12; Wiesler, Christine. (2020) ‘Epistemic Oppression and Ableism in Bioethics’ *Hypatia*, Vol. 35, p. 720.

8 Svenaeus, Fredrik. (2018) Phenomenological Bioethics: Medical Technologies, Human Suffering, and the Meaning of Being Alive. Routledge, London. p. 3; Leder, Drew. (1992) ‘Introduction’ in Leder, Drew (Ed) The Body in Medical Thought and Practice. Springer, p. 2; Toombs ‘The Meaning of Illness’, p. 227.

9 Leder, Drew. (1992) ‘Introduction’ p. 2; Leder Drew. (1984) ‘Medicine and Paradigms of Embodiment’ *The Journal of Medicine and Philosophy*, Vol. 9, p. 29; Rothfield, Philipa. (2005) ‘Attending to Difference: Phenomenology and Bioethics’ in in Shildrick, Margrit and Mykitiuk, Roxann (Eds*) Ethics of the Body: Postconventional Challenges*. The MIT Press, Cambridge MA. p. 31; Svenaeus (2018) *Phenomenological Bioethics* p. 80.

10 Svenaeus (2018) *Phenomenological Bioethics*, p. 86; Carel, Havi. (2016) *Phenomenology of Illness.* Oxford University Press, Oxford. p. 15.

11 Svenaeus (2018) *Phenomenological Bioethics*, p. 63.

12 Carel (2016) *Phenomenology of Illness*, p. 51.

13 Toombs (1987) ‘The Meaning of Illness’ p. 227.

14 Leder (1984) ‘Medicine and Paradigms of Embodiment’ p. 36; Rothfield (2005) ‘Attending to Difference’ p. 32.

15 Svenaeus (2018) *Phenomenological Bioethics* p. 43, 70.

16 Ibid, p. 82.

17 Leder (1992) ‘Introduction’ p. 3.

18 Leder (1984) ‘Medicine and Paradigms of Embodiment’ p. 36.

19 Svenaeus (2018) *Phenomenological Bioethics* p. 80; Carel (2016) *Phenomenology of Illness*, p. 74.

20 Ibid, p. 35; Leder, (1992) ‘A Tale of Two Bodies’ p. 30.

21 Wendell, Susan. (1996) *The Rejected Body: Feminist Reflections on Disability*. Routledge, New York. p. 123.

22 Scully, Jackie Leach. (2008) ‘Moral Bodies: Epistemologies of Embodiment’ in Londemann, Hilde et al. (Eds) *Naturalized Bioethics*. Cambridge University Press, Cambridge. p. 24; Ouelette, Alicia. (2011) *Bioethics and Disability: Toward a Disability-conscious Bioethics.* Cambridge, Cambridge University Press. p. 324; Kaplan, Deborah. (2000) ‘The Definition of Disability: Perspective of the Disabled *Community’ Journal of Health Care Law and Policy*, Vol. 3, p. 353.

23 Shildrick, Margrit. (2015) ‘’Why Should Our Bodies End at the Skin?’’: Embodiment, Boundaries, and Somatechnics’ *Hypatia*, Vol. 30, No. 1, p. 14; Barnes, Elizabeth. (2014) ‘Valuing Disability, Causing Disability’ *Ethics*, Vol. 125, p. 89; Silvers, Anita. (2003) ‘People with Disabilities’ in LaFollette, Hugh (Ed) *The Oxford Handbook of Practical Ethics*. Oxford University Press, Oxford. p. 304.

24 Garland Thomson, Rosemary. (2017) Disability Bioethics: From Theory to Practice’ *Kennedy Institute of Ethics Journal*, Vol. 27, No. 2, pp. 327; Vehmas, Simo and Shakespeare, Tom. (2014) ‘Disability, Harm, and the Origins of Limited Opportunities’ *Cambridge Quarterly Healthcare Ethics*, Vol. 23, p. 41; Ouelette (2011) *Bioethics and Disability*’ p. 58; Cox-White, Becky and Boxall, Susanna Flavia. (2009) ‘Redefining Disability: Maleficent, Unjust and Inconsistent’ *Journal of Medicine and Philosophy*, Vol. 33, p. 558; Wendell (1996*) The Rejected Body*, p. 63.

25 Weiss, Gail. (2015) ‘The normal, the natural, and the normative: A Merleau-Pontian legacy to feminist theory, critical race theory, and disability studies’ *Continental Philosophy Review*, Vol. 48, p. 89; Shakespeare, Tom. (2014) *Disability Rights and Wrongs Revisited*. Routledge, Abingdon. p. 171; Longmore, Paul K. (1995) ‘Medical Decision-Making and People with Disabilities: A Clash of Cultures’ Journal of Law, Medicine, and Ethics, Vol. 23, p. 82.

26 Wendell, Susan. (1989) ‘Toward a Feminist Theory of Disability’ *Hypatia*, Vol. 4, No. 2, p. 111; Wendell, (1996) *The Rejected Body*, p. 94.

27 Ouelette (2011) *Bioethics and Disability*, p. 24.

28 Garland Thomson, Rosemary. (1997) *Extraordinary Bodies: Figuring Physical Disability in American Culture and Literature.* Columbia University Press, NYC. P. 6; Wendell (1996) *The Rejected Body*, p. 42; Silvers, Anita. (1995) ‘Reconciling Equality to Difference: Caring (F)or Justice for People with Disabilities’ *Hypatia*, Vol. 10, p. 47.

29 Koch, Tom. (2004) ‘The Difference that Difference Makes: Bioethics and the Challenge of *Disability’ Journal of Medicine and Philosophy*, Vol. 29, p. 710; Silvers (1995) ‘Reconciling Equality to Difference’ p. 47.

30 Ouelette (2011) *Bioethics and Disability*, p. 15; Barnes, Elizabeth. (2016) *The Minority Body: A Theory of Disability*. Oxford University Press, Oxford. p. 130.

31 Campbell, Stephen et al. (2021) ‘Disability and the Goods of Life’ *The Journal of Medicine and Philosophy*, Vol. 46, p. 704.

32 Shakespeare, (2014) Disability Rights and Wrongs Revisited. p. 154; Wendell (1996) The Rejected Body, p. 94.

33 Clayton, Janine. (2016) ‘Studying Both sexes: a guiding principle for biomedicine’ *The FASEB Journal*, Vol. 30, p. 1; Sherwin, Susan. (1992) *No Longer Patient: Feminist Ethics and Healthcare*. Temple University Press, Philadelphia. p. 1.

34 Stychin, Carl. F (1998) ‘Body Talk: Rethinking Autonomy, Commodification and the Embodied Legal Self’ in Sheldon, Sally and Thomson, Michael (Eds) *Feminist Perspectives on Healthcare Law.* Cavendish Publishing, London. p. 214; Shildrick, Margrit. (2005) ‘Beyond the Body of Bioethics: Challenging the Conventions’ in Shildrick, Margrit and Mykitiuk, Roxanne. (2005) *Ethics of the Body: Postconventional Challenges.* The MIT Press, Cambridge MA. p. 15; Stanley, Liz and Wise, Sue. (1993*) Breaking Out Again: Feminist Ontology and Epistemology.* Routledge, London. p. 59.

35 Burkitt, Ian. (1999) *Bodies of Thought*, Sage, London. p. 90; Diprose, Rosalyn. (1994) *The Bodies of Women: Ethics, Embodiment, and Sexual Difference*. Routledge, London. p. vii; Warren, Virginia. (1989) ‘Feminist Directions in Medical Ethics’ *Hypatia*, Vol. 4, No. 2, p. 74.

36 Diprose (1994) The Bodies of Women p. 116; Jackson, Gabrielle. (2019) *Pain and Prejudice*. Piatkus, London. p. 292.

37 Jackson, Gabrielle. (2019) Pain and Prejudice. Piatkus, London. p. 292; Dusenbery, Maya. (2017) *Doing Harm: The truth About How Bad Medicine and Lazy Science Leave Women Dismissed, Misdiagnosed, and Sick*. Harper One, New York. p. 31.

38 Lee, Suk Kyeong. (2019) ‘Sex as an important biological variable in biomedical research’ *BMB Reports*, Vol. 51, pp. 167; Dusenbery (2017) *Doing Harm*, p. 9.

39 Jackson (2019) *Pain and Prejudice*, p. 198.

40 Regensteiner, Judith et al. (2020) ‘Sex as a Biological Variable: The Importance of Curriculum Development in the 21st Century’ *Journal of Women’s Health*, Vol. 29, p. 854.

41 Jackson (2019) *Pain and Prejudice*. p. 294.

42 Hoffman, Diane E. and Tarzian, Anita. (2001) ‘The Girl Who Cried Pain: a Bias Against Women in the Treatment of Pain’ *Journal of Law, Medicine, and Ethics*, Vol. 29, p. 13.

43 Jackson (2019) *Pain and Prejudice*, p. 7; Dusenbery (2017) *Doing Harm*, p. 114.

44 Jackson (2019) *Pain and Prejudice*, p. 10.

45 Jackson (2019) *Pain and Prejudice*, p. 15.

46 Hoffman and Tarzian (2001) ‘The Girl Who Cried Pain’ p. 14.

47 Dusenbery, Maya. (2017) *Doing Harm: The truth About How Bad Medicine and Lazy Science Leave Women Dismissed, Misdiagnosed, and Sick*. Harper One, New York. p. 9; Grob Gerald. (2011) ‘The Rise of Fibromyalgia in 20th Century America’ Perspectives in Biology and Medicine, Vol. 54, p. 417.

48 Dusenbery (2017) *Doing Harm*, p. 10; Malterud, Kirsti. (1999) ‘The (Gendered) Construction of Diagnosis: Interpretation of Medical Signs in Women Patients’ *Theoretical Medicine and Bioethics*, Vol. 20, p. 274; Kanaan, Richard A A. (2018) ‘Neurologists, Psychiatrists, and the Angry Patients they Share’ *The American Journal of Bioethics*, Vol. 18, No. 5, p. 22; Werner, Anne and Malterud, Kirsti. (2003) ‘It is hard work behaving as a credible patient: encounters between women with chronic pain and their *doctors’ Social Science and Medicine*, Vol. 27, p. 1409; Hannaford, Susan. (1985) *Living Outside Inside: A Disabled Woman’s Experience.* Canterbury Press, Berkley California. p. 7.

49 Dusenbery (2017) *Doing Harm*, p. 276; O’Leary, Diane. (2018) ‘Why Bioethics Should Be Concerned With Medically Unexplained Symptoms’ *The American Journal of Bioethics*, Vol. 18, No. 5, p. 12.

50 Pellegrino, Edmund. (2004) ‘Philosophy of Medicine and Medical Ethics: A Phenomenological Perspective’ in Khushf, G. (Ed) *Handbook of Bioethics*, Springer. p. 197; Richard Baron (1992) ‘Why Aren’t More Doctors Phenomenologists?’ in Leder (Ed) *The Body in Medical Thought and Practice*. Springer. p. 46; Cassell, Eric. (1992) ‘The Body of the Future’ in Leder (Ed) *The Body in Medical Thought and Practice*. Springer. p. 243.

51 Pellegrino (2004) ‘Philosophy of Medicine and Medical Ethics’, p. 183. Toombs, S. Kay. (2019) ‘The Healing Relationship: Edmund Pellegrino’s philosophy of the physician-patient encounter’ *Theoretical Medicine and Bioethics*, Vol. 40, p. 218.

52 Toombs (2019) ‘The Healing Relationship’, p. 218; Svenaeus. (2018) *Phenomenological Bioethics*, p. xi.

53 Badcott, David. (2005) ‘The Expert Patient: Valid Recognition or False Hope?*’ Medicine, Healthcare and Philosophy*, Vol. 8, p. 173; Carel, Havi and Kidd, Ian James. (2014) ‘Epistemic Injustice in healthcare: a philosophical analysis’ *Medicine, Healthcare and Philosophy*, Vol. 17, p. 535; Pellegrino (2004) ‘Philosophy of Medicine and Medical Ethics’ p. 194.

54 Leder, (1992) ‘A Tale of Two Bodies’, p. 32.

55 Carel (2016) Phenomenology of Illness, p. 46.

56 Cordier, Jean Francois. (2014) ‘The Expert Patient: towards a novel definition’ *European Respiratory Journal*, Vol. 44, p. 854; Badcott (2005) ‘The expert patient’, p. 175; Carel and Kidd (2014) ‘Epistemic injustice in healthcare’ p. 529.

57 Slatman, Jenny. (2014) ‘Multiple Dimensions of Embodiment in Medical Practices’ *Medicine, Healthcare and Philosophy*, Vol. 17, p. 549; Toombs (2019) ‘The Healing Relationship’, p. 218; Rothfield (2005) ‘Attending to Difference’, p. 30.

58 Leder (1992) ‘A Tale of Two Bodies’ p. 22.

59 Freeman, Lauren and Stewart, Heather. (2019) ‘Epistemic Microaggressions and Epistemic Injustices in Clinical Medicine’ in Sherman, Benjamin R. and Goguen, Stacey. (Eds) (2019) *Overcoming Epistemic Injustice: Social and Psychological Perspectives*. Rowman and Littlefield. p. 124; Groopman, Jerome. (2007) *How Doctors Think.* Houghton Mifflin, Boston. p. 15; Montgomery, Kathryn. (2006) *How Doctors Think.* Oxford University Press, Oxford. p. 51.

60 Stanley, Donald E. (2019) ‘The Logic of Medical Diagnosis: Generating and Selecting Hypothesis’ *Topoi*, Vol. 38, p. 437.

61 Jackson (2019) *Pain and Prejudice*, p. 9.

62 Oudshoorn, Nelly. (2020) Resilient Cyborgs: Living and Dying with Pacemakers and Defibrillators. Palgrave Macmillan, Singapore. p. 5.

63 Ibid, p. 79.

64 Besmer, Kirk. (2012) ‘Embodying a Translation Technology: The Cochlear Implant and Cyborg Intentionality’ *Techne: Research in Philosophy and Technology*, Vol. 16, No. 3, p. 302.

65 Ibid, p. 303.

66 Ibid, p. 303; Finlay, Linda and Molano-Fisher, Patricia. (2008) ‘‘Transforming’ self and world: a phenomenological study of a changing lifeworld following a cochlear implant’ *Medicine, Healthcare and Philosophy*, Vol. 11, p. 260; Hallerg, Lillemor R M and Ringdahl, Anders. (2004) ‘Living with Cochlear Implants: experiences of 17 adult patients in Sweden’ *International Journal of Audiology*, Vol. 43, No. 2, pp. 119; Maki-Torkko, Elina et al. (2015) ‘From isolation and dependence to autonomy – expectations before and experiences after cochlear implantation in adult cochlear implant users and their significant others’, *Disability and Rehabilitation*, Vol. 37, No. 6, p. 543.

67 Watson, Victoria; Verschuur, Carl; and Lathlean, Judith. (2016) ‘Exploring the experiences of teenagers with cochlear implants’ *Cochlear Implants International*, Vol. 17, No. 6, p. 298; Finlay and Molano-Fisher (2008) ‘’Transforming’ self and world’, p. 261.

68 Fricker, Miranda. (2007) Epistemic Injustice: Power and the Ethics of Knowing’ OUP, Oxford. p. 1.

69 Ibid, p. 1.

70 Ibid, p. 1; Carel and Kidd (2014) ‘Epistemic injustice in healthcare’ p. 531; Carel (2016) *Phenomenology of Illness*, p. 183.

71 Carel and Kidd (2014) ‘Epistemic injustice in healthcare’ p. 173.

72 Carel, Havi and Kidd, Ian James. (2017) ‘Epistemic Injustice in Medicine and Healthcare’ in Kidd, Ian James; Medina, Jose; Pohlhaus, Gaile. (Eds) (2017) *The Routledge Handbook of Epistemic Injustice*. Routledge, Abingdon. p. 340.

73 Kidd, Ian James and Carel, Havi. (2017) ‘Epistemic Injustice and Illness’ *Journal of Applied Philosophy*, Vol. 34, No. 2, p. 178.

74 Freeman and Stewart (2019) ‘Epistemic Microaggressions and Epistemic Injustices in Clinical Medicine’ p. 126.

75 Jackson (2019) *Pain and Prejudice*, p. 11; Hoffman and Tarzian. (2001) ‘The Girl Who Cried Pain’ p. 13.

76 Cumberlege J. (2020) First do no harm: the report of the Independent Medicines and Medical Devices Safety Review. Available at: https://www.immdsreview.org.uk/Report.html Para 1.12.

77 Cumberlege Review (2020) Para 1.19.

78 Slatman (2014) ‘Multiple Dimensions of Embodiment in Medical Practices’ p. 551.

79 Conelius, Jaclyn (2015) ‘A woman’s experience: living with an implantable cardioverter defibrillator’ *Applied Nursing Research*, Vol. 28, p. 195; Gilbert, Frederic; Goddard, Eliza; Viaña, John Noel M; Carter, Adrian and Horne, Malcolm. (2017) ‘I Miss Being Me: Phenomenological Effects of Deep Brain Stimulation’ *AJOB Neuroscience*, Vol. 8, No. 2, p. 106; Baron (1992) ‘Why Aren’t More Doctors Phenomenologists?’, p. 46.

80 Blain-Moraes, Stefanie et al. (2012) ‘Barriers to and mediators of brain-computer interface user acceptance: focus group findings’ *Ergonomics*, Vol. 55, No. 2, pp. 517; Murray, C.D. (2004) ‘An Interpretative Phenomenological Analysis of the Embodiment of Artificial Limbs’ *Disability and Rehabilitation*, Vol. 26, No. 16, p. 964; Carel and Kidd (2014) ‘Epistemic injustice in healthcare’ p. 537.

81 Flear (2019) ‘Epistemic Injustice as a Basis for Failure?’ p. 706.

82 Baggot, Rob; Allsop, Judith; Jones, Kathryn. (2004) ‘Representing the repressed? Health consumer groups and the national policy process’ *Policy & Politics*, Vol. 32, No. 3, p. 327; Heinelt, Hubert. (2018) ‘Introduction’ in Heinelt, Hubert (Ed) Handbook on Participatory Governance’ Edward Elgar Publishing, Cheltenham. p. 4.

83 Von Hippel, Eric. (2005) *Democratising Innovation.* The MIT Press, Cambridge MA. p. 23; Anderson, Sarah P et al. (2021) ‘Exploring the perspectives of prosthetic and orthotic users: past and present experiences and insights for the future’ *Disability and Rehabilitation*, Vol. 44, No. 18, p. 5285; Hafner, Brian et al. (2016) ‘Characterizing mobility from the prosthetic limb user’s perspective: Use of focus groups to guide development of the Prosthetic Limb Users Survey of Mobility’ *Prosthetics and Orthotics International*, Vol. 40, No. 5, pp. 582; Specker Sullivan, Laura et al. (2018) ‘Keeping Disability in Mind: A Case Study in Implantable Brain-Computer Interface Research’ *Science & Engineering Ethics*, Vol. 24, p. 480.

84 Allsop, Judith et al. (2004) ‘Health Consumer groups in the UK: a new social movement?’ Sociology of Health and Illness, Vol. 26, No. 2, p. 740; Baggot, Allsop, and Jones (2004) ‘Representing the repressed?’ p. 318.

85 Brown, Phil et al. (2004) ‘Embodied Health Movements: New approaches to social movements in health’ *Sociology of Health and Illness*, Vol. 26, No. 1, p. 50.

86 Ibid, p. 57.

87 Roberts, Joseph T F et al. (2021) ‘Prescribing unapproved medical devices? The case of DIY artificial Pancreas systems’ Medical Law International, Vol. 21, p. 42.

88 Crabtree, T et al. (2019) ‘DIY Artificial Pancreas Systems: Here to Stay?’ Practical Diabetes, Vol. 35, pp. 63–68.

89 Medtronic, (2023) ‘Minimed 780G Product Page’. Available at: https://www.medtronic-diabetes.co.uk/insulin-pump-therapy/minimed-780 g-system (Accessed 28.03.23).

90 Tandem (2023) ‘T:Slim Product Page’. Available at: https://www.tandemdiabetes.com/en-gb/home (Accessed 28.03.23).

91 For further information on both DIY artificial pancreas systems see: Lewis, Dana. (2019) ‘History and Perspectives on DIY Closed Looping’ Journal of Diabetes Science and Technology, Vol. 13, pp. 790–793; Roberts, Joseph T F et al. (2021) ‘Prescribing unapproved medical devices? The case of DIY artificial Pancreas systems’ Medical Law International, Vol. 21, pp. 42–68; Jennings, Peter and Hussain, Sufyan (2020) ‘Do-It-Yourself Artificial Pancreas systems: A review of the Emerging Evidence and Insights from Healthcare Professionals’ Journal of Diabetes Science and Technology, Vol. 14, pp. 868–877.

92 Toombs (2019) ‘The Healing Relationship’, p. 222.

93 Flear (2019) ‘Epistemic Injustice as a Basis for Failure?, p. 695.

94 Cumberlege Review (2020) Para 1.1.

95 Ibid, Para 1.2.

96 Ibid, Para 1.16.i.

97 Ibid, Para 2.3.

98 Ibid, Para 2.3.

99 Ockenden D. (2022) Findings, conclusions and essential actions from the Independent Review of Maternity Services at the Shrewsbury and Telford Hospital NHS Trust. Available at: https://www.gov.uk/government/publications/final-report-of-the-ockenden-review p. 107; Sibley, Miles. (2022) ‘Ockenden report: the refusal of our healthcare service to take patient experience seriously’ BMJ, Paper: 2022;377:o875, p. 1.

100 Ockenden report (2020) p. 11.

101 Ibid, p. 117.

102 Ibid, p. ix; p. 89, p. 103.

103 Ibid, p. 43; Dobson, Juliet. (2022) ‘Ignoring women’s experience led to the NHS’s biggest maternity scandal’ *BMJ*, Paper: 2022;377:o899, p. 1; Sibley (2022) ‘Ockenden report: the refusal of our healthcare service to take patient experience seriously’ p. 1.

104 Cupples, Laura. (2020) ‘Disability, Epistemic Harms, and the Quality Adjusted Life Year’ International Journal of Feminist Approaches to Bioethics, Vol. 13, No. 1, pp. 45–62.

105 Scully, Jackie Leach. (2018) ‘From ‘She would say that, wouldn’t she?’ to ‘Does she take sugar?’ Epistemic Injustice and Disability’ *International Journal of Feminist Approaches to Bioethics*, Vol. 11, No. 1, p.110; MacKenzie, Catriona and Scully, Jackie Leach. (2007) ‘Moral Imagination, Disability, and Embodiment’ *Journal of Applied Philosophy*, Vol. 24, p. 335.

106 Mackenzie and Scully (2007) ‘Moral Imagination, Disability, and Embodiment’ p. 337; Carell (2016) *Phenomenology of Illness*, p. 10.

107 Nelson, Ryan H et al. (2022) ‘Bioethics and the Moral Authority of Experience’ *The American Journal of Bioethics*, Vol. 23, No. 1, pp. 12–24.

108 Lichfield, Paul. (2014) Independent Review of the Work Capability Assessment – Year 5’ p. 9. Available at: https://www.gov.uk/government/publications/work-capability-assessment-independent-review-year-5 (Accessed 28.03.23).

109 Department for Work and Pensions, (2016) ‘A Guide to Employment and Support Allowance – The Work Capability Assessment’ ESA214. p. Available at: Available at: https://citizen-network.org/uploads/attachment/409/the-peoples-review-of-the-wca.pdfhttps://assets.publishing.service.gov.uk/government/uploads/system/uploads/attachment_data/file/535942/esa214-july-2016.pdf (Accessed 28.03.23).

110 Gulland, Jackie. (2011) ‘Ticking Boxes: Decision-Making in Employment and Support Allowance’ *Journal of Social Security Law’*, vol. 18, No. 2, p. 71.

111 Gullard ‘Ticking Boxes’ p. 76.

112 Allridge, Peter. (2019) ‘On Being Able to Walk 20 m: The Introduction of Personal Independence Payments’ *Journal of Law and Society*, Vol. 46, No. 3 p. 465; Kennedy, Steven et al. (2017) ‘Work Capability Assessments’ House of Commons Library Debate Pack. DCP-2017-0254. p. 10.

113 Anonymous, (2012) ‘The People’s Review of the Work Capability Assessment’. Available at: https://citizen-network.org/uploads/attachment/409/the-peoples-review-of-the-wca.pdf (Accessed 28.03.23).

114 Kennedy (2017) ‘Work Capability Assessments’, p. 14.

115 Kennedy, Steven. (2015) ‘Introduction of Personal Independence Payment’ House of Commons Library Briefing Paper, No. 06861. p. 3.

116 Harris, Neville. (2014) ‘Welfare Reform and the Shifting Threshold of Support for Disabled People’ *The Modern Law Review*, Vol. 77, No. 6, p. 927.

117 Gray, Paul. (2017) ‘The Second Independent Review of the Personal Independence Payment Assessment’ DWP, p. 12.

118 Ibid, p. 41.

119 Allridge (2019) ‘On Being Able to Walk 20 Meters’, p. 468.

120 Barclay, Linda. (2011) ‘Justice and Disability: What Kind of Theorizing is Needed?’ *Journal of Social Philosophy*, Vol. 42, No. 3, p. 276; Bianchin, Matteo and Heylighen, Ann. (2018) ‘Just Design’ *Design Studies*, Vol. 54, p. 2; Newell, Alan and Gregor, Peter. (2000) ‘‘User Sensitive Inclusive Design’ In Search of a New Paradigm’ CUU’00: Proceedings on the 2000 Conference on Universal Usability’ p. 42.

121 Bianchin and Heylighen (2018) ‘Just Design’, p. 2.

122 Shakespeare (2014) *Disability Rights and Wrongs Revisited*, p. 37; Bianchin and Heylighen (2018) ‘Just Design’ p. 2.

123 Bianchin and Heylighen (2018) ‘Just Design’, p. 9.

124 Paul, Laurie Ann. (2014) Transformative Experience. Oxford University Press, Oxford. p. 59; Levy, Neil. (2002) ‘Reconsidering Cochlear Implants: The Lessons of Martha’s Vineyard’ Bioethics, Vol. 16, p. 135; Blume, Stuart. (1999) ‘Histories of Cochlear Implantation’ Social Science & Medicine, Vol. 49, p. 1263; Reuzel, Rob. (2004) ‘Interactive Technology Assessment of Paediatric Cochlear Implantation’ Poiesis & Praxis, Vol. 2, p. 120.

125 Levy (2002) ‘Reconsidering Cochlear Implants’ p. 141; Hallberg, Lillemor R. M. and Ringdahl, Anders. (2004) ‘Living with cochlear implants: experiences of 17 adult patients in Sweden’ International Journal of Audiology, Vol. 43, p. 116; Mäki-torkko, Elina Margaretta et al. (2015) ‘From isolation and dependence to autonomy – expectations before and experiences after cochlear implantation in adult cochlear implant users and their significant others’ Disability and Rehabilitation, Vol. 37, p. 542; Rapport, Frances et al. (2020) ‘Adult’s cochlear implant journeys through care: a qualitative study’ BMC Health Services Research, Vol. 20, 457 p. 4.

126 Levy (2002) ‘Reconsidering Cochlear Implants’ p. 144; Sparrow, Robert. (2005) ‘Defending Deaf Culture: The Case of Cochlear Implants’ *The Journal of Political Philosophy*, Vol. 13, p. 136.

127 Levy (2002) ‘Reconsidering Cochlear Implants’ p. 150; Sparrow (2005) ‘Defending Deaf Culture’ p. 151.

128 Shakespeare (2014) Disability Rights and Wrongs Revisited, p. 37.

129 I would like to thank an anonymous reviwer for Monash Bioethics Review for suggesting this solution to me.

130 Clarkson, P John; and Coleman, Roger. (2015) ‘History of Inclusive Design in the UK’ *Applied Ergonomics*, Vol. 46, p. 240.

131 Finlayson, James Gordon. (2005) *Habermas: A Very Short Introduction.* Oxford University Press, Oxford. p. 35.

132 Young, Iris Marion. (2002) *Inclusion and Democracy*. Oxford University Press, Oxford. p. 24.

133 Chick, Matthew. (2021) ‘The Epistemic Value of Testimony’ *Contemporary Political Theory*, Vol. 21, p. 94; Khosrowi, Donal & Reiss, Julian. (2019) ‘Evidence-Based Policy: The Tension Between the Epistemic and the Normative’ Critical Review, Vol. 31, p. 180.

134 Shakespeare (2014) Disability Rights and Wrongs Revisited. p. 63.

135 Harris (2014) ‘Welfare Reform and the Shifting threshold of Support for Disabled People’ p. 926.

136 Fricker (2007) *Epistemic Injustice*, p. 1.

137 Ansell, Chris and Gash, Alison. (2007) ‘Collaborative Governance in Theory and Practice*’ Journal of Public Administration Research and Theory*, Vol. 18, p. 556.

138 Brown, Mark. (2006) ‘Survey Article: Citizen Panels and the Concept of Representation’ *The Journal of Political Philosophy*, Vol. 14, p. 213; Chick, Matthew. (2021) ‘The Epistemic Value of Testimony’ *Contemporary Political Theory*, Vol. 21, p. 103; Young, Iris Marion. (1990) *Justice and the Politics of Difference*. Princeton University Press, Princeton. p. 94.

139 Blume, Stuart. (2017) ‘In search of experiential knowledge’ *Innovation*, Vol. 30, p. 92; Krick, Eva. (2022) ‘Citizen experts in participatory governance: Democratic and epistemic assets of service user involvement, local knowledge, and citizen science’ *Current Sociology*, Vol. 70, p. 1003; Young (1990) *Justice and the Politics of Difference*. p. 186.

140 Baggot, Allsop, and Jones (2004) ‘Representing the repressed?’ p. 321; Min, John B. (2016) ‘Inclusion and Epistemic Benefits of Deliberation’ *Contemporary Pragmatism*, Vol. 13, p. 53.

141 Borkman, Thomasina. (1976) ‘Experiential Knowledge: A New Concept for the Analysis of Self-Help Groups’ *Social Service Review*, Vol. 50, p. 446.

142 Charlton, James I. (2000) *Nothing About Us Without Us: Disability, Oppression, and Empowerment*. University of California Press, Berkley. p. 141.

143 Palmquist, Ben. (2020) ‘Equity, Participation, and Power: Achieving Health Justice Through Deep Democracy’ *The Journal of Law, Medicine, and Ethics*. Vol. 48, p. 399.

144 Many healthcare organisations in the UK already include service users on their policy committees including NICE, the British Medical Association, the Royal College of Obstetricians and Gynaecologists, the British Pain Society, the Royal College of General Practitioners, as well as many Clinical Commissioning Groups.

145 Krick (2022) ‘Citizen experts in participatory governance’ p. 1003; Ansell and Gash (2007) ‘Collaborative Governance in Theory and Practice’ p. 544.

146 Young (2000) *Inclusion and Democracy*. p. 142.

147 Palmquist (2020) ‘Equity, Participation, and Power: Achieving Health Justice Through Deep Democracy’ p. 399.

148 Fisher, Frank. (2012) ‘Participatory Governance from Theory to Practice’ in Levi-Faur, David (Ed) *The Oxford Handbook of Governance*. Oxford University Press, Oxford. p. 465; Palmquist (2020) ‘Equity, Participation, and Power: Achieving Health Justice Through Deep Democracy’, p. 406.

149 Oughton, Deborah. (2004) ‘The Promises and Pitfalls of Participation’ *Global Bioethics*, Vol. 17, No. 1, p. 187.

150 Irvin, Renee and Stansbury, John. (2004) ‘Citizen Participation in Decision-making: Is it Worth the Effort?’ *Public Administration Review*, Vol. 64, p. 59; Krick, Eva. (2022) ‘Participatory Governance Practices at the Democracy-Knowledge-Nexus’ *Minerva*, Vol. 60, p. 474; Ansell and Gash (2007) ‘Collaborative Governance in Theory and Practice’ p. 556; Arnstein, Sherry R. (1969) ‘A Ladder of Citizen Participation’ *Journal of the American Institute of Planners*, Vol. 35, No. 4., p. 217; Yishai, Yael. (2012) ‘Participatory Governance in Public Health: Choice, but no Voice’ in Levi-Faur, David (Ed) *The Oxford Handbook of Governance*. Oxford University Press, Oxford. p. 527.

151 Fricker (2007) *Epistemic Injustice*, p. 66.

152 Ibid, p. 89.

153 Ibid, p. 92.

154 Ibid, p. 22.

155 Mackenzie and Scully (2007) ‘Moral Imagination, Disability, and Embodiment’ p. 347.

156 Freeman, Lauren and Stewart, Heather. (2018) ‘Microaggressions in Clinical Medicine’ *Kennedy Institute of Ethics Journal*, Vol. 28, No. 4, p. 425.

157 Carel and Kidd (2014) ‘Epistemic injustice in healthcare’, p. 532.

158 Havi Carel, (2012) ‘Phenomenology as a Resource for Patients’ *The Journal of Medicine and Philosophy*, Vol. 37, No. 2, pp. 96–113.

159 Kidd and Carel (2014) ‘Epistemic injustice in healthcare’, p. 537.

160 Kidd and Carel (2017) ‘Epistemic Injustice and Illness’ p. 187.

161 Carel (2012) ‘Phenomenology as a Resource for Patients’ p. 107.

162 Ibid, p. 108.

163 Ibid, p. 108.

164 Ibid, p. 109.

165 Wisnewski, J. Jeremy. (2015) ‘Perceiving Sympathetically: Moral Perception, Embodiment, and Medical Ethics’ *Journal of Medical Humanities*, Vol. 36, pp. 309–319.

166 Sorial, Sarah. (2022) ‘Deliberation and the Problems of Exclusion and Uptake: The Virtues of Actively Facilitating Equitable Deliberation and Testimonial Sensibility’ *Ethical Theory and Moral Practice*, Vol. 25, p. 215.

167 Fricker (2007) *Epistemic Injustice.* p. 81; Sorial (2022) ‘Deliberation and the Problems of Exclusion and Uptake’ p. 215.

168 Wisnewski (2015) ‘Perceiving Sympathetically’ p. 318; Benner, Patricia. (2000) ‘The role of embodiment, emotion and lifeworld for rationality and agency in nursing practice’ *Nursing Philosophy*, Vol. 1, p. 14; Olufowote, James Olumide. (2015) ‘Virtue Training in Medical Schools: The Perspective of Behavioural Science Course Directors’ *Health Communication*, Vol. 30, No. 4, p. 363; Pellegrino, Edmund and Thomasma, Pellegrino, Edmund and Thomasma, David C. (1993) *Virtues in Medical Practice.* New York, Oxford Uni Press. p. 178.

169 Fricker (2007) *Epistemic Injustice* p. 159.

170 Ibid. 158.

171 Ibid. p. 148; Kidd, Ian James and Carel, Havi. (2019) ‘Pathocentric Epistemic Injustice and Conceptions of Health’ in Sherman, Benjamin R and Gouguen, Stacey (Eds) *Overcoming Epistemic Injustice: Social and Psychological Perspectives.* Rowman and Littlefield. p. 158.

172 Flear (2019) ‘Epistemic Injustice as a Basis for Failure?’ p. 713: Vicari, Stefania and Cappai, Franco. (2016) ‘Health activism and the logic of connective action. A case study of rare disease patients organisations’ Information, *Communication, and Society*, Vol. 19, No. 1, pp. 1656; Brown et al. (2004) ‘Embodied health movements’ p. 54.

173 Fricker (2007) *Epistemic Injustice* p.169.

174 Ibid p. 168–169.

175 Medina, Jose. (2012) ‘Hermeneutical injustice and Polyphonic Contextualism: Social Silences and shared Hermeneutical Responsibilities’ *Social Epistemolog*y, Vol. 26, No. 2, p. 216.

176 Medina, Jose. (2017) ‘Varieties of Hermeneutic Injustice’ in in Kidd, Ian James; Medina, Jose; Pohlhaus, Gaile. (Eds) (2017) *The Routledge Handbook of Epistemic Injustice*. Routledge, Abingdon. p. 48.

177 Medina (2017) ‘Varieties of Hermeneutic Injustice’ p. 48; Fricker (2007) *Epistemic Injustice* p. 172.

