# Supplementary material for: Taking embodiment seriously in public policy and practice: adopting a procedural approach to health and welfare
Source: Monash Bioeth Rev. 2023 Nov 4;41(Suppl 1):20–48. doi: 10.1007/s40592-023-00183-x (PMC10754762; doi:10.1007/s40592-023-00183-x)
Supplement: Supplementary file 1 — Supplementary Material 1 [file 40592_2023_183_MOESM1_ESM.docx]

Response to Reviewers – ‘Taking Embodiment Seriously in Public Policy and Practice: Adopting a Procedural Approach to Health and Welfare’ (MOBR-D-23-00009)

Comment:

This is an excellent paper, providing an overview of an important, and often neglected, issue in our approach to healthcare. It breaks new ground in the discussion of this issue in trying to provide concrete guidance on how to take embodiment seriously in healthcare policy. While the solution suggested is necessarily sketchy, given the amount of ground the paper covers, I don't think that's a problem in this instance.

There's just one place where I think there is some oversimplification going on. On pp18-19 the author discusses the tensions around the use of cochlear implants, and concludes as a result that "there is not even the potential for dissolving the tension in this case." But that's not actually true in reality. It would be possible to allow CIs, but only in parallel with a policy fostering the teaching and uptake of sign language (for example). And in practice, what has happened is a softening of the resistance of the Deaf community as it has become clearer that CIs don't inevitably render sign language pointless. Both of these points might be examples of solutions that could come out of the participatory process the author suggests.

I look forward to seeing this paper published.

Response:

First of all, thank you for the kind review. In response to your comment that I have oversimplified the case on pg 18, I have removed the suggestion that there is no way of dissolving the tension between CI use and the promotion of Deaf culture. Instead, I have moved the example of potential tensions between CI and Deaf culture up to the previous paragraph where I discuss tensions which may be resolvable with sufficient imagination.

The suggestion that we could dissolve the tension between CI and deaf culture by allowing CI implantation only on the condition that we also have a policy of promoting the uptake and use of sign language has been included into the body of the text and a footnote thanking the anonymous reviewer for the observation has been added.
